# A generalized optimization principle for asymmetric branching in fluidic networks

**DOI:** 10.1098/rspa.2016.0451

**Published:** 2016-07

**Authors:** David Stephenson, Duncan A. Lockerby

**Affiliations:** School of Engineering, University of Warwick, Coventry CV4 7AL, UK

**Keywords:** Murray's law, asymmetric branching, optimization principle, biomimetic, non-Newtonian fluids, turbulent flows

## Abstract

When applied to a branching network, Murray’s law states that the optimal branching of vascular networks is achieved when the cube of the parent channel radius is equal to the sum of the cubes of the daughter channel radii. It is considered integral to understanding biological networks and for the biomimetic design of artificial fluidic systems. However, despite its ubiquity, we demonstrate that Murray’s law is only optimal (i.e. maximizes flow conductance per unit volume) for symmetric branching, where the local optimization of each individual channel corresponds to the global optimum of the network as a whole. In this paper, we present a generalized law that is valid for asymmetric branching, for any cross-sectional shape, and for a range of fluidic models. We verify our analytical solutions with the numerical optimization of a bifurcating fluidic network for the examples of laminar, turbulent and non-Newtonian fluid flows.

## 1. Introduction

The optimal branching of fluidic networks has been the subject of numerous studies owing to its importance in understanding the behaviour of biological vessels and for the biomimetic design of artificial systems. Much of the research stems from Murray’s law [1], who posited that there were two main energy requirements for blood flow through a cylindrical vessel of radius *R*: (i) the energy required to overcome viscous drag and drive the flow and (ii) the energy required to metabolically maintain the fluid and vessel. Assuming the flow to be laminar, Newtonian, steady, incompressible and fully developed, Murray used the Hagen–Poiseuille equation to model the driving power requirement (i.e. the driving power is proportional to *R*^−4^), and assumed that the maintenance power was proportional to the volume of the channel (i.e. proportional to *R*^2^). Applying the principle of minimum work to the total power requirement, Murray surmised that in an optimized cylindrical channel, the volumetric flow rate *Q* is proportional to *R*^3^. By applying mass conservation over a branching network, and assuming that local pressure losses through the junction (owing to bends and channel contractions) are negligible compared with the pressure losses over the channel lengths, this principle is most commonly expressed as a power law between a parent channel and *N* daughter channel branches

1.1 $$R_{p}^{3}=\sum_{i=1}^{N}R_{d_{i}}^{3},$$

where the subscripts p and d_*i*_ denote the parent and *i*th daughter, respectively. Although originally targeting blood transport through the cardiovascular system [2–9], experimental data have shown Murray’s law to be a decent approximation for a number of other biological networks, e.g. in the bronchial trees of humans and dogs [10–12]; in the chick embryo [13], and in the leaf veins of plants [14–16].

Whole blood (plasma and cells) is a non-Newtonian fluid that exhibits shear-thinning behaviour, i.e. its viscosity decreases with increased shear-strain rate. To more accurately model vascular networks, Murray’s law has been applied to non-Newtonian fluids [17,18] using the popular power-law fluid model [19,20]. In both studies, it was found that the optimal radius relation is unaffected by shear-thinning or shear-thickening fluid behaviour, and equation (1.1) is maintained for the whole range of non-Newtonian fluids. Murray’s law has also been applied to turbulent flows [21–23], which can be found in the upper airways of the lung [24] and, under some circumstances, in blood flow through the aorta [25], as well as in a number of hydraulic and pneumatic civil engineering applications. For fully rough-wall turbulent flow, the flow rate was found to be proportional to *R*^7/3^, leading to the relation

1.2 $$R_{p}^{7/3}=\sum_{i=1}^{N}R_{d_{i}}^{7/3},$$

for branching networks.

While Murray’s law has been most often applied to circular or elliptical [18] cross sections (owing to the shape of biological networks), optimized branching is also useful for the design of artificial systems [5], such as for fuel cells [26] or heat exchangers [27,28], which are often constrained to certain shapes by manufacturing procedures. To this end, Murray’s law has been adapted for networks of rectangular and trapezoidal cross sections [29].

Although originally derived from the principle of minimum work, it has been noted that the application of other optimization principles results in the same relationship between flow rate and channel radius: minimizing the total mass of the channel [22], minimizing volume for a constant pressure drop and flow rate [30], minimizing pumping power [31], maintaining a constant shear stress in all channels [32], and minimizing flow resistance for a constant volume [5,23,33].

However, despite many developments to Murray’s law, we submit that it is, in fact, suboptimal for asymmetric branching. In this paper, we derive a generalized law that is applicable to symmetric and asymmetric branching, for any cross-sectional shape, and for a range of fluidic models (e.g. Newtonian and non-Newtonian, laminar and turbulent).

## 2. Analytical solutions

The conditions for optimal branching can be generalized as a maximization of flow conductance per unit volume through each branch of the network, for a variety of constraint combinations. As with Murray’s law, we assume the flow to be steady and fully developed. For a two-level network (consisting of a single parent channel branching into multiple daughter channels), this can be expressed as

2.1 $${\arg\;\max}_{\Gamma_{j}\in [0,\infty ]}\left[\frac{Q}{\Delta PV}\right]\;\mathrm{subject}\;\mathrm{to}\;\mathrm{fixed}\;\left\{ \begin{array}{l} Q,\Delta P\\ V,\Delta P\\ V,Q, \end{array}\right.$$

where *Q* is the volumetric flow rate through the parent channel, $\Delta P={\left\{\Delta P_{i}\right\}}_{i=1}^{N}$ is the set of pressure drops Δ*P*_*i*_ over each of the *N* network branches—from the inlet of the parent to the outlet of the corresponding daughter—*V* is the network volume, and

2.2 $$\Gamma_{j}=\frac{A_{d_{j}}}{A_{p}}$$

is the *j*th daughter–parent cross-sectional area ratio. Note, for each constraint option, two parameters are fixed and the third parameter is optimized (volume minimization, flow-rate maximization and pressure-drop minimization, respectively). Therefore, all three constraint options lead to an identical optimal relation because

2.3 $$\frac{dV}{d\Gamma_{j}}=\frac{dQ}{d\Gamma_{j}}=\frac{d(\Delta P)}{d\Gamma_{j}}=0$$

regardless of the constraints chosen. Note, noting that the last term means that d(Δ*P*_*i*_)/d*Γ*_*j*_=0 for all *i*. For our optimization, we assume that the channel lengths *L* are large compared with the size of the parent–daughters junction, so that (i) the localized pressure losses at the junction are negligible compared with the pressure drops over individual channels (as in Murray’s law), and (ii) the volume of the network can be considered to be the sum of the channel volumes

2.4 $$V=A_{p}L_{p}+\sum_{i=1}^{N}A_{d_{i}}L_{d_{i}}.$$

If we consider the optimization of the *j*th daughter channel, then inserting equation (2.4) into the fitness function of equation (2.1), and noting equation (2.3), gives

2.5 $$\frac{d}{d\Gamma_{j}}\left(\frac{\Delta PV}{Q}\right)=L_{p}\frac{dA_{p}}{d\Gamma_{j}}+\sum_{i=1}^{N}L_{d_{i}}\frac{dA_{d_{i}}}{d\Gamma_{j}}=0$$

The pressure drop over the parent and each of the *i* daughter channels can be expressed in terms of the volumetric flow rate

2.6 $$\Delta P_{p}=QL_{p}k_{p}$$

and

2.7 $$\Delta P_{d_{i}}=\Psi_{i}QL_{d_{i}}k_{d_{i}},$$

where *Ψ*_*i*_=*Q*_d_*i*__/*Q* is the fraction of the total flow rate taken by the *i*th daughter channel and *k* is flow resistance per unit length, e.g. *k*_p_=Δ*P*_p_/(*QL*_p_). The pressure drop over each network branch Δ*P*_*i*_=Δ*P*_p_+Δ*P*_d_*i*__ is then

2.8 $$\Delta P_{i}=Q(L_{p}k_{p}+\Psi_{i}L_{d_{i}}k_{d_{i}}).$$

Differentiating equation (2.8) with respect to *Γ*_*j*_, and noting equation (2.3), gives

2.9 $$L_{p}\frac{dk_{p}}{d\Gamma_{j}}+\Psi_{i}L_{d_{i}}\frac{dk_{d_{i}}}{d\Gamma_{j}}=0.$$

Substituting equation (2.9) into equation (2.5), via the chain rule, gives our generalized optimal area ratio

2.10 $${\frac{dA}{dk}|}_{p}={\sum_{i=1}^{N}\frac{1}{\Psi_{i}}\frac{dA}{dk}|}_{d_{i}},$$

which, for brevity, will be referred to as the *generalized law* to distinguish it from Murray’s law. It should be noted that the subscript *j* is not present in equation (2.10), so this relationship is not specific to a particular daughter channel; it relates the properties of *all* daughter channels to that of the parent.

This generalized law is valid for any cross-sectional shape, for any fluid (e.g. non-Newtonian) and for any Reynolds number (e.g. for turbulent flow). It is also valid for flows through nanoscale networks where the fluid is dominated by velocity slip at the walls; however, in the paper, we restrict our attention to the continuum-flow limit. We now consider some important cases where *A* can be expressed easily as an analytical function of *k*.

### (a) Laminar flow

The steady-state incompressible Navier–Stokes momentum equation describes laminar flow through a long channel with an arbitrary cross-sectional shape, i.e.

2.11 $$\frac{\Delta P}{L}=-\mu \nabla^{2}u,$$

where *μ* is the dynamic viscosity (constant for a Newtonian fluid) and *u* is the streamwise channel velocity. This can be non-dimensionalized using Δ*P*/*L*, *μ* and cross-sectional area *A*, such that

2.12 $$1=-{\tilde{\nabla }}^{2}\tilde{u},$$

where

2.13 $$u=\tilde{u}A\left(\frac{\Delta P}{L}\right)\frac{1}{\mu };\;\nabla^{2}=\frac{{\tilde{\nabla }}^{2}}{A},$$

and tilde denotes a dimensionless quantity or operator. The axes of the cross-sectional plane are defined as *y*,*z* and

2.14 $$y=\tilde{y}\sqrt{A};\;z=\tilde{z}\sqrt{A}.$$

Provided the boundary conditions are fixed (which is the case for the continuum-flow limit, where the no-slip boundary condition applies), the solution of equation (2.12), $\tilde{u}(\tilde{y},\tilde{z})$, is independent of *A*, Δ*P*, *L* and *μ*, and is thus a property of the cross-sectional shape alone. Similarly, so is

2.15 $$S=\iint_{A}\tilde{u}(\tilde{y},\tilde{z})\;d\tilde{y}\;d\tilde{z}.$$

An expression for the volumetric flow rate is obtained by integrating the fluid momentum over the cross-sectional area

2.16 $$Q=\iint_{A}u\;dy\;dz.$$

Substitution of equations (2.13)–(2.15) into (2.16) gives the volumetric flow rate through a channel with an arbitrary cross-sectional shape

2.17 $$Q=A^{2}\left(\frac{\Delta P}{L}\right)\frac{S}{\mu },$$

and flow resistance per unit length

2.18 $$k=\frac{\mu }{SA^{2}}.$$

It is assumed that the pressure drop over the network is small such that the viscosity and density are constants for a Newtonian fluid. For cylindrical channels *S*=1/(8*π*), equation (2.17) becomes the Hagen–Poiseuille flow rate. If the cross-sectional shape is constant throughout the network, i.e. *S*=const., substituting (2.18) into the generalized law (2.10) and cancelling the constant terms gives

2.19 $$A_{p}^{3}=\sum_{i=1}^{N}\frac{1}{\Psi_{i}}A_{d_{i}}^{3}.$$

From equation (2.7), it can be seen that

2.20 $$\frac{\Delta P_{d_{i}}}{\Psi_{i}L_{d_{i}}k_{d_{i}}}=\mathrm{const}.$$

for all daughter channels. Combining equations (2.18) and (2.20) produces the cross-sectional area relationship between the *i*th and *j*th daughter channels

2.21 $$A_{d_{i}}=A_{d_{j}}\sqrt{\frac{\Psi_{i}\Phi_{ij}}{\Psi_{j}}},$$

where

2.22 $$\Phi_{ij}=\frac{\left(\Delta P_{d_{j}}/L_{d_{j}}\right)}{\left(\Delta P_{d_{i}}/L_{d_{i}}\right)}$$

is the pressure-gradient ratio between the *j*th and *i*th daughter channels. Note that, because the shape property *S* cancels in equation (2.21), the generalized law will be independent of the cross-sectional shape of the channels. Substituting equation (2.21) into equation (2.19) and rearranging for *Γ*_*j*_ as defined by equation (2.2) gives

2.23 $$\Gamma_{j}=\sqrt{\Psi_{j}}{\left[\sum_{i=1}^{N}\Phi_{ij}\sqrt{\Psi_{i}\Phi_{ij}}\right]}^{-1/3}.$$

Equation (2.23) relates the area of the parent channel to the area of the *j*th daughter channel in an optimized two-level network of laminar flow. It is valid for any cross-sectional shape, provided the shape is constant through the network. Equation (2.23) is only equivalent to Murray’s law (which is $\Gamma_{j}=\Psi_{j}^{2/3}$ when posed in terms of an area ratio) if the daughter channels branch symmetrically, i.e. *Ψ*_*i*_=*Ψ*_*j*_=1/*N* and *Φ*_*ij*_=1. By inserting these constraints into equation (2.23), the symmetric generalized law for laminar flow is

2.24 $$\Gamma =N^{-2/3}.$$

This means that for symmetric branching, Murray’s law is valid for any cross-sectional shape, not just circles. The reason Murray’s law produces a suboptimal result for asymmetric branching is that it was derived to optimize a single channel in isolation. However, as shown above (and verified later), for asymmetric branching, the global optimum is not the same as the optimum for each channel considered separately. One important reason for this is that the result of Murray’s single-channel optimization (*Q*∝*R*^3^) is independent of the pressure drop; so when applied to a branching network, the relative pressure drops over the daughter channels are not considered, and the optimization is under-constrained. Murray’s original principle, over time, has been misinterpreted as a general branching law (for symmetric *and* asymmetric configurations), leading to the prevalence of the incorrect form shown in equation (1.1). This misinterpretation has endured in subsequent literature regarding turbulent flow [21] and non-Newtonian fluids [17,18], as we shall now demonstrate.

### (b) Turbulent flow

Turbulent flow is described by the phenomenological Darcy–Weisbach equation, which relates the pressure drop to the mean velocity for an incompressible fluid in a channel of arbitrary cross-sectional shape

2.25 $$\Delta P=\frac{fL{\bar{u}}^{2}}{2D},$$

where *f* is the Darcy friction factor, $\bar{u}$ is the mean streamwise velocity, D=4A/P is the hydraulic diameter, and P is the wetted perimeter. Note that equation (2.25) is applicable to gravity-driven open channels, e.g. rivers, as well as closed pipes. In a river, the pressure drop is a function of the channel slope [23]. Making the substitutions $R=\sqrt{A}/P$ (which is a property of the cross-sectional shape, like *S*) and $Q=\bar{u}A$, equation (2.25) can be rewritten as

2.26 $$\Delta P=\frac{fLQ^{2}}{8RA^{5/2}},$$

and the flow resistance per unit length *k* is

2.27 $$k=\frac{fQ}{8RA^{5/2}}.$$

For turbulent flows, the pressure drop is proportional to the square of the volumetric flow rate, so *k* is a function of *Q*. However, for all constraint options of the optimization described by equation (2.1), d*Q*/d*Γ*_*j*_=0. For the sake of deriving an analytical expression comparable to Murray’s law, we restrict our interest to fully rough-wall turbulent flow, where the friction factor is also approximately constant^1^ —i.e. it is independent of the Reynolds number and the volumetric flow rate. In this regime, the main applications are civil engineering hydraulic and pneumatic systems. So, when the shape is constant through the network, substituting equation (2.27) into the generalized law (equation (2.10)) gives

2.28 $$A_{p}^{7/2}=\sum_{i=1}^{N}\frac{1}{\Psi_{i}^{2}}A_{d_{i}}^{7/2}.$$

Combining equations (2.20) and (2.27) produces the cross-sectional area relationship between the *i*th and *j*th daughter channels

2.29 $$A_{d_{i}}=A_{d_{j}}{\left(\frac{\Psi_{i}^{2}\Phi_{ij}}{\Psi_{j}^{2}}\right)}^{2/5}.$$

Substituting equation (2.29) into equation (2.28) and rearranging for the daughter–parent area ratio gives

2.30 $$\Gamma_{j}=\Psi_{j}^{4/5}{\left[\sum_{i=1}^{N}\Phi_{ij}{\left(\Psi_{i}^{2}\Phi_{ij}\right)}^{2/5}\right]}^{-2/7}.$$

Equation (2.30) relates the area of the parent channel to the area of the *j*th daughter channel in an optimized two-level network for turbulent flow. It is valid for channels of any cross-sectional shape, provided the shape is constant through the network, and is only equivalent to the turbulent Murray’s law [21] (equation (1.2)) for symmetric branching, i.e. *Ψ*_*i*_=*Ψ*_*j*_=1/*N* and *Φ*_*ij*_=1, where (2.30) reduces to

2.31 $$\Gamma =N^{-6/7}.$$

This also agrees with the turbulent flow symmetric branching results from previous studies [22,23]. Comparing equations (2.24) and (2.31) shows that, for symmetric branching, the optimal daughter–parent area ratio is smaller for turbulent flow than it is for laminar flow.

### (c) Non-Newtonian fluid flow

Non-Newtonian fluids are typically characterized by a nonlinear relationship between shear stress and shear-strain rate. The power-law constitutive model [19,20] is one of the most popular, enabling a wide range of engineering problems to be solved analytically. For fluid flow through a circular channel, this is

2.32 $$\tau =m{\left(\frac{du}{dr}\right)}^{n},$$

where *m* is the flow consistency index, d*d*/d*r* is the shear strain rate and *n* is the flow behaviour index. This relationship leads to an effective viscosity of

2.33 $$\mu =m{\left(\frac{du}{dr}\right)}^{n-1}.$$

Power-law fluids can be divided into three classes based on their flow behaviour index: (i) pseudo-plastic (shear thinning) fluids (*n*<1) exhibit a decrease in viscosity with increased shear strain rate; (ii) Newtonian fluids (*n*=1) exhibit a constant viscosity; and (iii) dilatant (shear thickening) fluids (*n*>1) exhibit an increase in viscosity with increased shear strain rate. Owing to the difficulty of applying the power-law model in two dimensions, the optimal branching of non-Newtonian fluids are considered here only for circular cross sections.

In cylindrical coordinates, the steady-state Navier–Stokes equation for incompressible laminar flow through a circular cross section is

2.34 $$\frac{\Delta P}{L}=-\frac{1}{r}\frac{d}{dr}\left(\mu r\frac{du}{dr}\right),$$

where *u* is the streamwise velocity (the radial and swirl velocity components are assumed to be zero). Substituting equation (2.33) into (2.34) and integrating with respect to *r* gives

2.35 $$\frac{\Delta Pr}{2L}=-m{\left(\frac{du}{dr}\right)}^{n}+C_{1},$$

where *C*_1_ is a constant. At the midpoint of the cross section, when *r*=0, the velocity is at a maximum and thus d*d*/d*r*=0; therefore, *C*_1_=0. Integrating with respect to *r* once more produces

2.36 $$u=-{\left(\frac{\Delta P}{2Lm}\right)}^{1/n}\left(\frac{n}{n+1}\right)r^{1+1/n}+C_{2},$$

where *C*_2_ is another constant. Inserting the no-slip condition at the wall into equation (2.36) produces the non-Newtonian velocity profile

2.37 $$u={\left(\frac{\Delta PR}{2Lm}\right)}^{1/n}\left(\frac{nR}{n+1}\right)\left[1-{\left(\frac{r}{R}\right)}^{1+1/n}\right].$$

The volumetric flow rate is obtained by integrating the fluid momentum over the cross-sectional area, which, in cylindrical coordinates, is

2.38 $$Q=\int_{0}^{R}\int_{0}^{2\pi }ur\;dr\;d\theta ,$$

where *θ* is the azimuth. Substituting equation (2.37) into (2.38) gives

2.39 $$Q={\left(\frac{\Delta PR}{2Lm}\right)}^{1/n}\left(\frac{n\pi R^{3}}{3n+1}\right).$$

By setting *n*=1 in equation (2.39) the Hagen–Poiseuille equation is recovered. Noting that *A*=*πR*^2^, the flow resistance per unit length is

2.40 $$k=\left[2m\pi^{\left(n+1\right)/2}{\left(\frac{3n+1}{n}\right)}^{n}Q^{n-1}\right]A^{-(3n+1)/2}.$$

For non-Newtonian fluids, there is a nonlinear relationship between the pressure gradient and the volumetric flow rate, so *k* is again a function of *Q*. As explained previously, d*Q*/d*Γ*_*j*_=0 for all the constraint options in the optimization described by equation (2.1), so substituting equation (2.40) into the asymmetric generalized law (equation (2.10)) gives

2.41 $$A_{p}^{\left(3n+3\right)/2}=\sum_{i=1}^{N}\left(\frac{1}{\Psi_{i}^{n}}\right)A_{d_{i}}^{\left(3n+3\right)/2}.$$

Combining equations (2.20) and (2.40) produces the cross-sectional area relationship between the *i*th and *j*th daughter channels

2.42 $$A_{d_{i}}=A_{d_{j}}{\left[{\left(\frac{\Psi_{i}}{\Psi_{j}}\right)}^{n}\Phi_{ij}\right]}^{2/\left(3n+1\right)}.$$

Substituting equation (2.42) into equation (2.41), and rearranging for the daughter–parent area ratio gives

2.43 $$\Gamma_{j}=\Psi_{j}^{2n/\left(3n+1\right)}{\left[\sum_{i=1}^{N}\Phi_{ij}{\left(\Psi_{i}^{n}\Phi_{ij}\right)}^{2/\left(3n+1\right)}\right]}^{-2/\left(3n+3\right)}.$$

Equation (2.43) relates the area of the parent channel to the area of the *j*th daughter channel in an optimized two-level network of circular channels transporting a non-Newtonian fluid. By setting *n*=1, the asymmetric generalized law for Newtonian fluid flows (equation (2.23)) is retrieved. Equation (2.43) shows that *Γ*_*j*_ is dependent on the flow behaviour index *n*, contrary to results from previous studies on non-Newtonian branching flows that used Murray’s law [17,18]. The optimal daughter–parent area ratio is independent of *n* only for symmetric branching, i.e. *Ψ*_*i*_=*Ψ*_*j*_=1/*N* and *Φ*_*ij*_=1:

2.44 $$\Gamma =N^{-2/3}.$$

This is exactly the same as equation (2.24) for symmetric Newtonian flows and agrees with previous studies [17,18] for symmetric non-Newtonian flows. Equation (2.43) can also be used to determine the optimal area ratio for the theoretical limits of the flow behaviour index *n*. For the shear-thickening-fluid limit, when n→∞, equation (2.43) reduces to

2.45 $$\Gamma_{j}=\Psi_{j}^{2/3}.$$

Interestingly, equation (2.45) is independent of the daughter–daughter pressure-gradient ratio *Φ*_*ij*_ and is exactly the same as Murray’s law for asymmetric branching (1.1) when posed in terms of areas. For the shear-thinning-fluid limit, when n→0, equation (2.43) reduces to

2.46 $$\Gamma_{j}={\left[\sum_{i=1}^{N}\Phi_{ij}^{3}\right]}^{-2/3}.$$

For this limit, the optimal area ratio is independent of the daughter flow-rate fraction *Ψ*_*j*_, so when the daughter channel pressure gradients are equal, i.e. *Φ*_*ij*_=1, the daughter channel areas are also equal and *Γ*=*N*^−2/3^.

## 3. Numerical verification and discussion

In this section, we construct a numerical model of a two-level branching network which adopts the same fluid-physics assumptions used in Murray’s original paper, its subsequent extensions for turbulent flows and non-Newtonian fluid flows, and our own generalized law. These are (i) the flow through each channel is steady-state, incompressible and fully developed; (ii) the pressure is continuous throughout the network; and (iii) the pressure linearly varies over the entire length of each channel from inlet/outlet to a common branching point. The purpose of using the same physical model for the fluidic network is solely to demonstrate that its optimization does not lead to Murray’s law, but to the generalized law we derived above; verification of Murray’s fluid-physics assumptions is beyond the scope of this paper.

For clarity, we present the numerical model in a form specific to laminar flow through cylindrical channels (as per Murray’s original case), and refer the reader to appendix A for a more general description. The flow through each channel of the fluidic network is determined by momentum conservation, and is treated as being positive if it flows towards the point of branching—i.e. flow through the parent channel will be positive and flow through daughters will be negative. In the specific case of laminar flow through a cylindrical channel, and given the previously stated fluid-physics assumptions, this is the Hagen–Poiseuille law

3.1 $$q_{i}=\frac{a_{i}^{2}}{8\pi \mu }\frac{\left(p_{i}-p_{B}\right)}{l_{i}},$$

where *q*_*i*_ is the volumetric flow rate through the *i*th channel in the network, *a*_*i*_ is the cross-sectional area of the *i*th channel, *p*_*i*_ is the pressure at the end of the *i*th channel (i.e. the inlet of the parent channel or the outlet of a daughter channel), *l*_*i*_ is the length of the *i*th channel and *p*_B_ is the pressure at the point of branching (which is common to all channels). Note, here, unlike in our analytical derivation, the subscript *i* could denote either the parent channel (*i*=1) or one of the daughter channels (*i*=2,3…,*M*, where *M* is the total number channels that comprise the network). The model is completed by mass continuity at the branching point, i.e.

3.2 $$\sum_{i=1}^{M}q_{i}=0,$$

calculation of the total network volume *v*, i.e.

3.3 $$v=\sum_{i=1}^{M}a_{i}l_{i},$$

and the definition of the cross-sectional area ratio between the (*i*+1)th and *i*th channels

3.4 $$\gamma_{i}=\frac{a_{i+1}}{a_{i}}.$$

The system of equations (3.1)–(3.4) can be solved for the mass flow rates *q*_(1:*M*)_ if the following are fixed: pressure at boundaries *p*_(1:*M*)_, channel lengths *l*_(1:*M*)_, fluid viscosity *μ*, network volume *v*, and the channel cross-sectional area ratios *γ*_(1:*M*−1)_. In this paper, the solution is obtained using the trust-region dogleg algorithm [34] in MATLAB${\;}^{®}$.

To enable a comparison with Murray’s law and our generalized law, we now optimize the network model (i.e. equations (3.1)–(3.4)) using a brute-force approach. For otherwise fixed properties (e.g. fixed volume, boundary pressures, etc.), the cross-sectional area ratio between the first daughter channel and the parent channel area *γ*_1_ is varied, through all physically viable values, to locate that which maximizes the volumetric flow rate through the network. This result can then be compared directly with Murray’s law and the generalized law, as the definition of *γ*_1_ is equivalent to that of *Γ*_d_1__.

### (a) Laminar flow

The first set of optimization results demonstrate that Murray’s law is suboptimal for asymmetrically branching networks of any cross-sectional shape, even for laminar flow. To demonstrate that our generalized law is valid for any cross-sectional shape (as long as the shape remains constant through the network), the numerical verification is performed for three different cross sections: circular, square, and rectangular with an aspect ratio *α*=5. For circles, *S*=1/(8*π*), and for rectangles, an accurate approximation of *S* is calculated from simulations, using a standard central-difference solution of the laminar Navier–Stokes equations (2.12).^2^ For Murray’s law, mass conservation provides the closure $R_{d_{i}}^{3}=R_{d_{j}}^{3}(1-\Psi_{j})/\Psi_{j}$, which leads to

3.5 $$\Gamma_{j}=\Psi_{j}^{2/3}.$$

**Figure 1.**
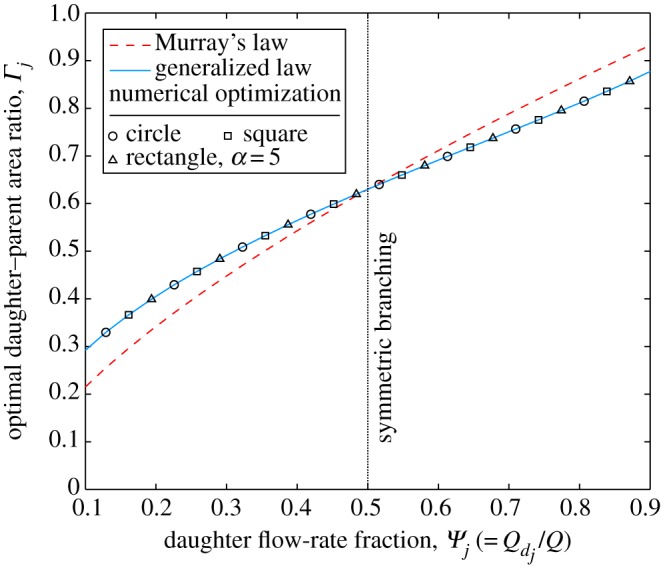
Optimal daughter–parent area ratio (*Γ*_*j*_) against daughter flow-rate fraction (*Ψ*_*j*_) for laminar flow through a two-level bifurcating network with equal daughter channel pressure gradients (*Φ*_*ij*_=1). Plotted for Murray’s law (equation (3.5)), the generalized law (equation (2.23)), and the results from the numerical optimization for circles, squares, and rectangles of aspect ratio *α*=5. (Online version in colour.)

In figure 1, to induce asymmetry, the daughter flow-rate fraction *Ψ*_*j*_ is varied whereas the daughter–daughter pressure-gradient ratio is kept constant at *Φ*_*ij*_=1. All solutions that the greater the fraction of flow through the daughter channel, the greater the optimum daughter’s area (relative to the parent); as is intuitive. The results confirm the finding that Murray’s law is only optimal for symmetric bifurcations (*Ψ*=0.5); for a flow-rate percentage of 10% (*Ψ*=0.1), Murray’s law under-predicts the optimum daughter area by as much as 26%. In contrast, the generalized law is accurate for all values of *Ψ*_*j*_ for all cross-sectional shapes tested. This confirms the analytical finding that Murray’s law has been mistakenly applied to asymmetrically branching networks, where the optimized result for each individual channel is not optimal for the network as a whole.

**Figure 2.**
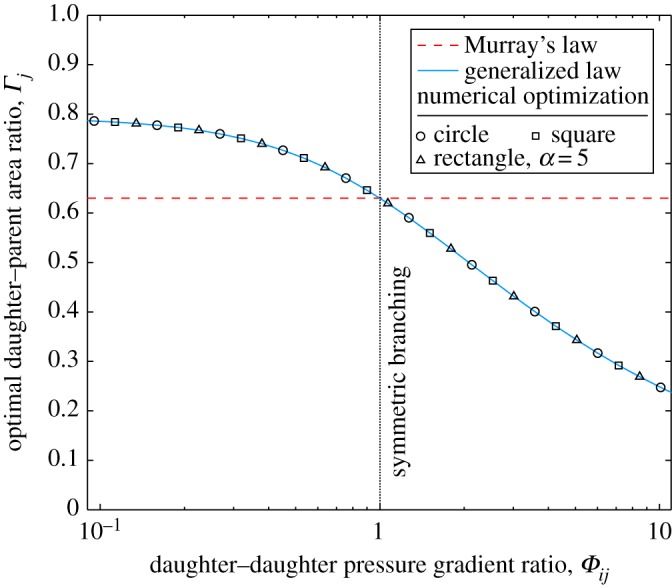
Optimal daughter–parent area ratio (*Γ*_*j*_) against daughter–daughter pressure-gradient ratio *Φ*_*ij*_=(Δ*P*_d_*j*__/*L*_d_*j*__)/(Δ*P*_d_*i*__/*L*_d_*i*__) for laminar flow through a two-level bifurcating network with equal flow through each daughter channel, i.e. *Ψ*_*j*_=*Ψ*_*i*_=0.5. Plotted for Murray’s law (equation (3.5)), the generalized law (equation (2.23)), and the results from the numerical optimization for circles, squares, and rectangles of aspect ratio *α*=5. (Online version in colour.)

This can be further demonstrated by inducing asymmetry by varying the daughter–daughter pressure-gradient ratio *Φ*_*ij*_, and maintaining a constant daughter flow-rate fraction *Ψ*_*j*_=0.5, as shown in figure 2. Murray’s law does not consider *Φ*_*ij*_ to be a variable that affects the optimal daughter–parent area ratio *Γ*_*j*_ and shows a notable departure from the numerical optimization results; for a pressure-gradient ratio of *Φ*_*ij*_=2, Murray’s law over predicts the optimum daughter area by 24%. In contrast, the generalized law is accurate for all values of *Φ*_*ij*_, for all cross-sectional shapes and, as expected, shows that the optimal area of the *j*th daughter channel is smaller when it has a larger pressure gradient relative to the other daughter channel, as the flow rate flowing through each daughter is equal (*Ψ*_*j*_=*Ψ*_*i*_=0.5). This result is the same whether the pressure gradient is altered by varying the relative daughter channel lengths or the pressure drops. Figures 1 and 2 both show that as the extent of asymmetry increases, Murray’s law provides a poorer estimate of the optimal area ratio.

### (b) Turbulent flow

The next set of optimization results are for fully rough-wall turbulent flow through an asymmetrically bifurcating network of channels with arbitrary, but constant, cross-sectional shape. For Murray’s law, the closure $R_{d_{i}}^{7/3}=R_{d_{j}}^{7/3}(1-\Psi_{j})/\Psi_{j}$ is provided by mass conservation, which leads to

3.6 $$\Gamma_{j}=\Psi_{j}^{6/7}.$$

**Figure 3.**
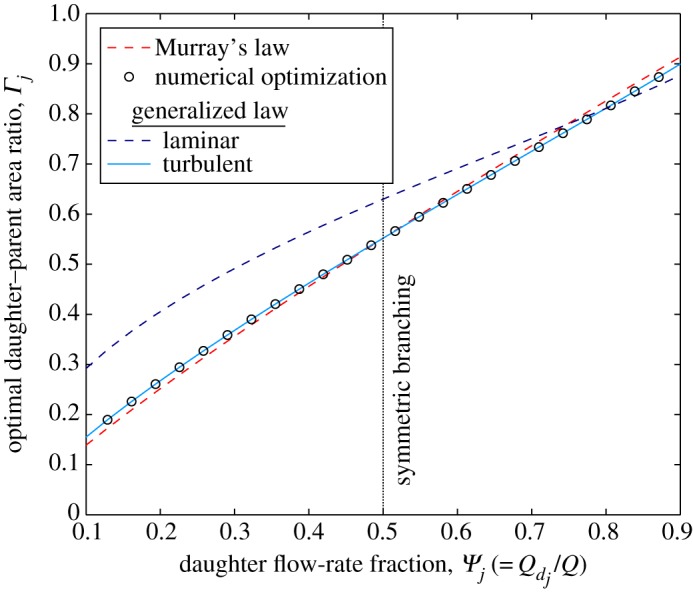
Optimal daughter–parent area ratio (*Γ*_*j*_) against daughter flow-rate fraction (*Ψ*_*j*_) for fully rough turbulent flow through a two-level bifurcating network of arbitrary, but constant, cross-sectional shape with equal daughter channel pressure gradients (*Φ*_*ij*_=1). Plotted for Murray’s law (equation (3.6)), the laminar generalized law (equation (2.23)), the turbulent generalized law (equation (2.30)), and results from the numerical optimization. (Online version in colour.)

**Figure 4.**
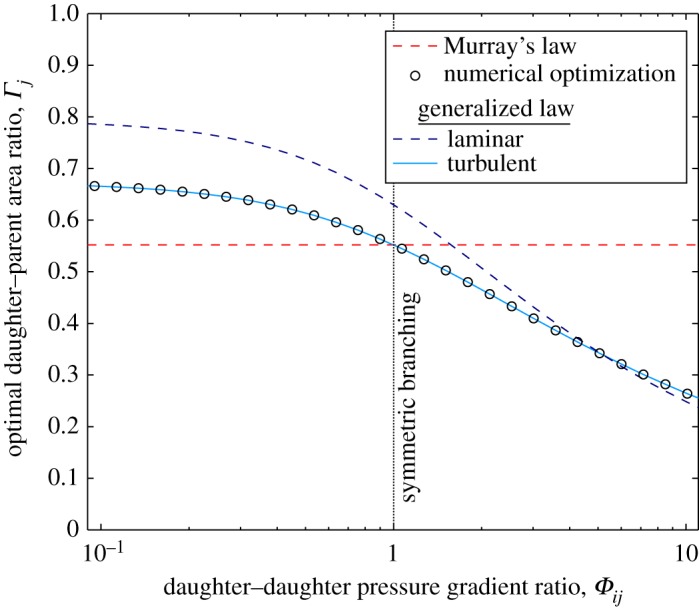
Optimal daughter–parent area ratio (*Γ*_*j*_) against daughter–daughter pressure-gradient ratio (*Φ*_*ij*_) for fully rough-wall turbulent flow through a two-level bifurcating network of arbitrary, but constant, cross-sectional shape. The volumetric flow rate through each daughter channel is equal, i.e. *Ψ*_*j*_= 0.5. Plotted for Murray’s law (equation (3.6)), the laminar generalized law (equation (2.23)), the turbulent generalized law (equation (2.30)), and results from the numerical optimization. (Online version in colour.)

The optimization results shown in figures 3 and 4 have asymmetry induced by varying the daughter flow-rate fraction *Ψ*_*j*_ and daughter–daughter pressure-gradient ratio *Φ*_*ij*_, respectively. Again, there is excellent agreement between the numerical optimization and the turbulent generalized law for all values of *Ψ*_*j*_ and *Φ*_*ij*_. Both figures 3 and 4 show that, except in the case of large asymmetries, the optimal daughter–parent area ratio for laminar flow is larger than the optimal area ratio for turbulent flow. This trend can broadly be explained by considering the equations for volumetric flow rate for laminar and turbulent flow (equations (2.17) and (2.26), respectively). For laminar flow *Q*∝*A*^2^, whereas for turbulent flow *Q*∝*A*^5/4^. Considering these relationships for the parent and the *j*th daughter channel, then $\Gamma_{j}\propto \Psi_{j}^{1/2}$ and $\Gamma_{j}\propto \Psi_{j}^{4/5}$ for laminar and turbulent flows, respectively (this is confirmed by the generalized laws for laminar flow (2.23) and turbulent flow (2.30)). As *Ψ*_*j*_ is always less than one, $\Psi_{j}^{1/2}>\Psi_{j}^{4/5}$ and thus the optimal area ratio for laminar flow will generally be larger than optimal area ratio for turbulent flow for the same fixed parameters.

Murray’s law proves to be a more accurate approximation for asymmetrically branching turbulent flows (compared with laminar flows), but still errs by 10% when *Ψ*_*j*_=0.1 (and *Φ*_*ij*_=1), and by 19% when *Φ*_*ij*_=2 (and *Ψ*_*j*_=0.5). For symmetric branching (*Ψ*=0.5 and *Φ*_*ij*_=1), the numerical and analytical solutions both agree with Murray’s law [21] and the results by [22,23].

### (c) Non-Newtonian fluid flow

**Figure 5.**
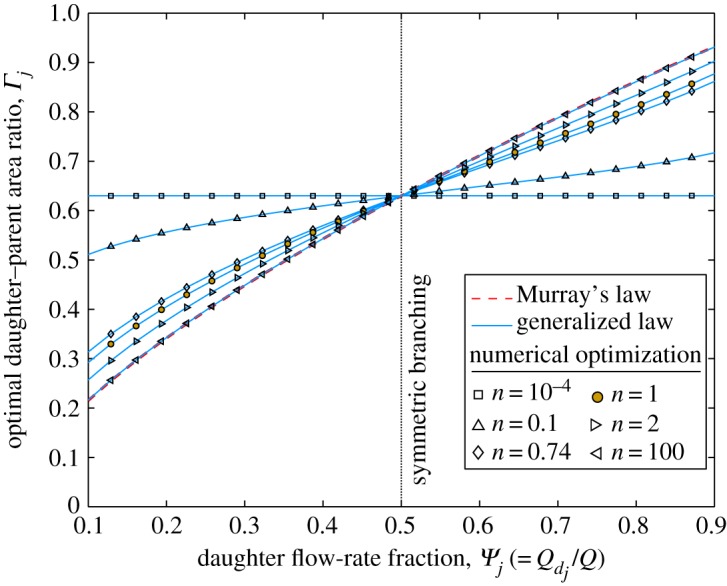
Optimal daughter–parent area ratio (*Γ*_*j*_) against daughter flow-rate fraction (*Ψ*_*j*_) in a two-level bifurcating network of circular channels with equal daughter channel pressure gradients (*Φ*_*ij*_=1). Comparison of Murray’s law (equation (3.5)), the generalized law (equation (2.43)), and results from the numerical optimization. Plotted for *n*=10^−4^, 0.1, 0.74 (blood), 1 (Newtonian fluid), 2 and 100. (Online version in colour.)

The final set of optimization results are for non-Newtonian fluid flow through an asymmetrically bifurcating network of circular channels. For Murray’s law, equation (3.5) is used. In figure 5, asymmetry is induced by varying *Ψ*_*j*_, whereas *Φ*_*ij*_=1 is constant. The results demonstrate that the optimal daughter–parent area ratio *Γ*_*j*_ is dependent on the flow behaviour index *n*, contrary to the results of previous studies based on Murray’s law [17,18].

**Figure 6.**
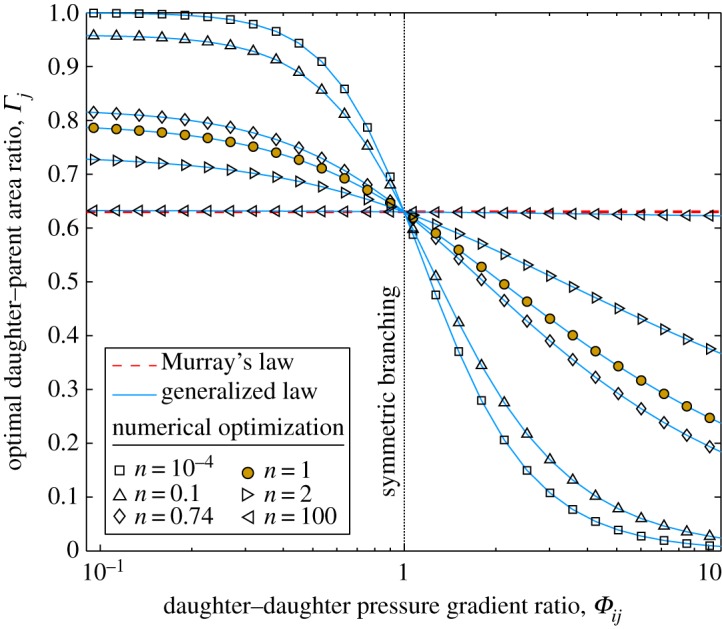
Optimal daughter–parent area ratio (*Γ*_*j*_) against daughter–daughter pressure-gradient ratio (*Φ*_*ij*_) in a two-level bifurcating network of circular channels with *Ψ*_*j*_=0.5. Comparison of Murray’s law (equation (3.5)), the generalized law (equation (2.43)), and results from the numerical optimization. Plotted for *n*=10^−4^, 0.1, 0.74 (blood), 1 (Newtonian fluid), 2, and 100. (Online version in colour.)

The Newtonian fluid case (*n*=1) is highlighted with a filled marker, and it is noted that this solution is the same as that shown in figure 1. It is observed that, for all *n*, the gradient (d*Γ*_*j*_/d*Ψ*_*j*_) increases monotonically with increasing *n*. As n→∞, the fluid approaches the shear-thickening-fluid limit (equation (2.45)) where Murray’s law is correct for all *Ψ*_*j*_. For smaller values of *n*, Murray’s law is correct only for symmetric bifurcations (*Ψ*_*j*_=0.5) and becomes increasingly inaccurate as *n* decreases; for a flow-rate percentage of 10% (*Ψ*_*j*_=0.1), Murray’s law under predicts *Γ*_*j*_ by 66% for *n*=10^−4^. The increasing error in the Murray’s law solution as *n* decreases is also shown in figure 6, where asymmetry is induced by varying *Φ*_*ij*_ and *Ψ*_*j*_=0.5 is fixed. Here, for a pressure-gradient ratio of *Φ*_*ij*_=2, Murray’s law over predicts the optimum daughter area by 172% when *n*=10^−4^. In contrast, the generalized law is accurate for all values of *Ψ*_*j*_ and *n*. The plot for *n*=0.74 is an approximation of the optimal area ratio for the cardiovascular system, based on the measurements of a falling-ball viscometer [35]. As n→0, the fluid approaches the shear-thinning-fluid limit (equation (2.46)) and *Γ*_*j*_ becomes independent of *Ψ*_*j*_.

The reason for the shear-thickening- and shear-thinning-fluid limits can be found by examining the volumetric flow rate of a non-Newtonian fluid. When n→0, by raising all terms to the power of *n*, equation (2.39) becomes 1=Δ*PR*/(2*Lm*) and the area ratio is only a function of the pressure gradient; hence, *Γ*_*j*_ does not vary with *Ψ*_*j*_. When the daughter–daughter pressure-gradient ratio *Φ*_*ij*_ decreases, the area must increase (and vice versa), as shown in figure 6. Conversely, when n→∞, equation (2.39) becomes *Q*=*πR*^3^/3 and the optimal area ratio is only a function of the volumetric flow rate; hence, *Γ*_*j*_ does not vary with *Φ*_*ij*_. This expression, with *Q*∝*R*^3^, is equivalent to Murray’s law (equation (1.1)), where the local optimization is the same as the global optimization, as shown in figures 5 and 6.

## 4. Conclusion

We have derived a generalized optimization principle that leads to analytical expressions for the optimum daughter–parent area ratio *Γ* for asymmetrically branching networks of any cross-sectional shape and for a range of fluidic models. This new optimal relation will enable deeper understanding of biological network behaviour and provide a generalized biomimetic design principle that can be applied to a variety of artificial branching systems to maximize their efficiency.

We have verified analytical solutions using a numerical optimization procedure and shown that, for symmetric branching of laminar and Newtonian fluids, our generalized law is equivalent to Murray’s law. However, when applied to an asymmetrically branching network, Murray’s law is suboptimal, as the global optimization of the entire network is not equal to the local optimization of each individual channel, which Murray’s law presumes. We further demonstrate that this mistake in the application of Murray’s law to asymmetric branching networks has endured for non-Newtonian fluids (e.g. in the cardiovascular system) and turbulent flows (e.g. in hydraulic or pneumatic civil engineering applications).

In non-Newtonian fluidic networks, *Γ* is dependent on the flow behaviour index *n* for asymmetric branching, contrary to what previous studies based on Murray’s law have stated. Murray’s law is only retrieved for non-Newtonian fluid networks at the shear-thickening limit, when n→∞ and *Γ* is no longer dependent on the relative pressure gradients over the daughter channels. At the shear-thinning limit, when n→0, *Γ* becomes independent of the relative flow rates through each daughter channel.

## Footnotes

1 A solution for the whole range of Reynolds numbers can be obtained by using an empirical relation between the friction factor and the Reynold’s number, e.g. fitting to the Moody diagram.

2 Our numerical solver is an in-house code written in MATLAB${\;}^{®}$, which uses matrix inversion to calculate the volumetric flow rates. All simulations use a 100×100 mesh, which has been shown (via a grid resolution study) to provide volumetric flow rate solutions to within 1% of the values obtained using a 200×200 and 300×300 mesh.

## Appendix A. General volumetric flow rate expression for the numerical optimization procedure

The equation for volumetric flow rate through the *i*th straight channel can be generally expressed as

A 1 $$q_{i}=\mathrm{sgn}(p_{i}-p_{B})b_{1}a_{i}^{b_{2}}{\left(\frac{p_{i}-p_{B}}{l_{i}}\right)}^{b_{3}},$$

where *b*_1_, *b*_2_ and *b*_3_ are flow-model-dependent constants. Table 1 shows the values of the constants for laminar flow through a channel of arbitrary cross-sectional shape, fully rough-wall turbulent flow through a channel of arbitrary cross-sectional shape, and non-Newtonian fluid flow through a channel with a circular cross section.

**Table 1.** Volumetric flow rate constants for laminar flow, fully rough-wall turbulent flow, and non-Newtonian flow.

flow model	*b*_1_	*b*_2_	*b*_3_
laminar	SμAA	2	1
turbulent	8RfAAAAA	$\frac{5}{4}$	$\frac{1}{2}$
non-Newtonian	n(3n+1)12mπ(n+1)/2nAAAAAA	$\frac{\left(3n+1\right)}{2n}$	$\frac{1}{n}$

## References

[1] Murray CD 1926 The physiological principle of minimum work: I. the vascular system and the cost of blood volume. *Proc. Natl Acad. Sci. USA* 12, 207–214. (doi:10.1073/pnas.12.3.207) 16576980 10.1073/pnas.12.3.207 PMC1084489

[2] Hutchins GM, Miner MM, Boitnott JK 1976 Vessel caliber and branch-angle of human coronary artery branch-points. *Circ. Res.* 38, 572–576. (doi:10.1161/01.RES.38.6.572) 1269108 10.1161/01.res.38.6.572

[3] Kassab GS, Fung YB 1995 The pattern of coronary arteriolar bifurcations and the uniform shear hypothesis. *Ann. Biomed. Eng.* 23, 13–20. (doi:10.1007/BF02368296) 7762878 10.1007/BF02368296

[4] LaBarbera M 1990 Principles of design of fluid transport systems in zoology. *Science* 249, 992–1000. (doi:10.1126/science.2396104) 2396104 10.1126/science.2396104

[5] Sherman TF 1981 On connecting large vessels to small. the meaning of Murray’s law. *J. Gen. Physiol.* 78, 431–453. (doi:10.1085/jgp.78.4.431) 7288393 10.1085/jgp.78.4.431 PMC2228620

[6] Zamir M, Brown N 1982 Arterial branching in various parts of the cardiovascular system. *Am. J. Anat.* 163, 295–307. (doi:10.1002/aja.1001630403) 7091015 10.1002/aja.1001630403

[7] Zamir M, Medeiros JA 1982 Arterial branching in man and monkey. *J. Gen. Physiol.* 79, 353–360. (doi:10.1085/jgp.79.3.353) 7077288 10.1085/jgp.79.3.353 PMC2215753

[8] Zamir M, Medeiros JA, Cunningham TK 1979 Arterial bifurcations in the human retina. *J. Gen. Physiol.* 74, 537–548. (doi:10.1085/jgp.74.4.537) 512630 10.1085/jgp.74.4.537 PMC2228563

[9] Zamir M, Wrigley SM, Langille BL 1983 Arterial bifurcations in the cardiovascular system of a rat. *J. Gen. Physiol.* 81, 325–335. (doi:10.1085/jgp.81.3.325) 6842176 10.1085/jgp.81.3.325 PMC2215579

[10] Wilson TA 1967 Design of the bronchial tree. *Nature* 213, 668–669. (doi:10.1038/213668a0) 6031769 10.1038/213668a0

[11] Horsfield K, Cumming G 1967 Angles of branching and diameters of branches in the human bronchial tree. *Bull. Math. Biophys.* 29, 245–259. (doi:10.1007/BF02476898) 6051603 10.1007/BF02476898

[12] Horsfield K, Relea FG, Gumming G 1976 Diameter, length and branching ratios in the bronchial tree. *Respir. Physiol.* 26, 351–356. (doi:10.1016/0034-5687(76)90005-0) 951538 10.1016/0034-5687(76)90005-0

[13] Taber LA, Ng S, Quesnel AM, Whatman J, Carmen CJ 2001 Investigating Murray’s law in the chick embryo. *J. Biomech.* 34, 121–124. (doi:10.1016/S0021-9290(00)00173-1) 11425071 10.1016/s0021-9290(00)00173-1

[14] McCulloh KA, Sperry JS, Adler FR 2003 Water transport in plants obeys Murray’s law. *Nature* 421, 939–942. (doi:10.1038/nature01444) 12607000 10.1038/nature01444

[15] McCulloh KA, Sperry JS, Adler FR 2004 Murray’s law and the hydraulic vs mechanical functioning of wood. *Funct. Ecol.* 18, 931–938. (doi:10.1111/j.0269-8463.2004.00913.x)

[16] McCulloh KA, Sperry JS 2005 The evaluation of Murray’s law in *Psilotum nudum* (Psilotaceae), an analogue of ancestral vascular plants. *Am. J. Bot.* 92, 985–989. (doi:10.3732/ajb.92.6.985) 21652482 10.3732/ajb.92.6.985

[17] Revellin R, Rousset F, Baud D, Bonjour J 2009 Extension of Murray’s law using a non-Newtonian model of blood flow. *Theor. Biol. Med. Modell.* 6, 7 (doi:10.1186/1742-4682-6-7) 10.1186/1742-4682-6-7 PMC2695432 19445663

[18] Tesch K 2010 On some extensions of Murray’s law. *Task Q.* 14, 227–235.

[19] Ostwald W 1925 About the rate function of the viscosity of dispersed systems. *Kolloid Z* 36, 99–117. (doi:10.1007/BF01431449)

[20] de-Waele A 1923 Viscometry and plastometry. *Oil. Color. Chem. Assoc. J.* 6, 33–88.

[21] Uylings H 1977 Optimization of diameters and bifurcation angles in lung and vascular tree structures. *Bull. Math. Biol.* 39, 509–520. (doi:10.1007/BF02461198) 890164 10.1007/BF02461198

[22] Williams HR, Trask RS, Weaver PM, Bond IP 2008 Minimum mass vascular networks in multifunctional materials. *J. R. Soc. Interface* 5, 55–65. (doi:10.1098/rsif.2007.1022) 17426011 10.1098/rsif.2007.1022 PMC2605499

[23] Bejan A, Rocha LAO, Lorente S 2000 Thermodynamic optimization of geometry: T- and y-shaped constructs of fluid streams. *Int. J. Therm. Sci.* 39, 949–960. (doi:10.1016/S1290-0729(00)01176-5)

[24] Olsen DE, Dart GA, Filley GF 1970 Pressure drop and fluid flow regime of air inspired into the human lung. *J. Appl. Physiol.* 28, 482–494. 5437439 10.1152/jappl.1970.28.4.482

[25] Stein PD, Sabbah HN 1976 Turbulent blood flow in the ascending aorta of humans with normal and diseased aortic valves. *Circ. Res.* 39, 58–65. (doi:10.1161/01.RES.39.1.58) 776437 10.1161/01.res.39.1.58

[26] Senn SM, Poulikakos D 2004 Laminar mixing, heat transfer and pressure drop in tree-like microchannel nets and their application for thermal management in polymer electrolyte fuel cells. *J. Power Sources* 130, 178–191. (doi:10.1016/j.jpowsour.2003.12.025)

[27] Chen Y, Cheng P 2002 Heat transfer and pressure drop in fractal tree-like microchannel nets. *Int. J. Heat Mass Transf.* 45, 2643–2648. (doi:10.1016/S0017-9310(02)00013-3)

[28] Wang X, Mujumdar AS, Yap C 2006 Thermal characteristics of tree-shaped microchannel nets for cooling of a rectangular heat sink. *Int. J. Therm. Sci.* 45, 1103–1112. (doi:10.1016/j.ijthermalsci.2006.01.010)

[29] Emerson DR, Cieślicki K, Gu X, Barber RW 2006 Biomimetic design of microfluidic manifolds based on a generalised Murray’s law. *Lab. Chip* 6, 447–454. (doi:10.1039/b516975e) 16511629 10.1039/b516975e

[30] Kamiya A, Togawa T 1972 Optimal branching structure of the vascular tree. *Bull. Math. Biophys.* 34, 431–438. (doi:10.1007/BF02476705) 4659270 10.1007/BF02476705

[31] Gosselin L, Bejan A 2005 Tree networks for minimal pumping power. *Int. J. Therm. Sci.* 44, 53–63. (doi:10.1016/j.ijthermalsci.2004.06.004)

[32] Zamir M 1977 Shear forces and blood vessel radii in the cardiovascular system. *J. Gen. Physiol.* 69, 449–461. (doi:10.1085/jgp.69.4.449) 853286 10.1085/jgp.69.4.449 PMC2215050

[33] Bejan A 2000 *Shape and structure, from engineering to nature*. Cambridge, UK: Cambridge University Press.

[34] Powell MJD 1970 A Fortran subroutine for solving systems of nonlinear algebraic equations. In *Numerical methods for nonlinear algebraic equations* (ed. P Rabinowitz), ch. 7. Philadelphia, PA: Gordon and Breach Science Publishers.

[35] Eguchi Y, Karino T 2008 Measurement of rheologic property of blood by a falling-ball blood viscometer. *Ann. Biomed. Eng.* 36, 545–553. (doi:10.1007/s10439-008-9454-7) 18259867 10.1007/s10439-008-9454-7

